# Preliminary study of the association between corneal histocytological changes and surgically induced astigmatism after phacoemulsification

**DOI:** 10.1186/1471-2415-14-134

**Published:** 2014-11-20

**Authors:** Xing Du, Guiqiu Zhao, Qing Wang, Xian Yang, Ang Gao, Jing Lin, Qian Wang, Qiang Xu

**Affiliations:** Department of Ophthalmology, the Affiliated Hospital of Medical College of Qingdao University(AHMCQU), NO. 16 Jiangsu Road, 266003 Qingdao, Shandong province China

**Keywords:** Cataract, Phacoemulsification, Corneal endothelium, Corneal keratocytes, Confocal microscopy, SIA

## Abstract

**Background:**

Surgically induced astigmatism (SIA) was one of the factors that influences the desirable refractive outcome, and it was related to the length, type, location, structure of the incision and to the suture closure technique, etc. The aim was to evaluate the association of corneal histocytological changes with SIA after phacoemulsification.

**Methods:**

The study enrolled 68 cases of cataract patient (68 eyes). Corneal histocytological parameters at corneal incision, central cornea and contralateral incision obtained by confocal microscope through focusing (CMTF) were compared preoperatively and 1 week, 2 weeks, 1 month, 3 months and 6 months postoperatively. These biometric parameters included the endothelial cell density, keratocyte density of posterior stromal layer, and the morphological changes. SIA was calculated by Jaffe’s vector analysis.

**Results:**

1 From preoperatively to 1 week, 2 weeks, 1 month, 3 months and 6 months postoperatively, the endothelail cell density was decreased significantly (p < 0.05). Keratocyte density of posterior stroma layer was increased significantly only at 1 week, 2 weeks, 1 month, 3 months postoperatively (p <0.05), but not statistically significant (p = 0.173) at 6 months postoperatively compared to preoperative values. 2 The histocytological observations indicated that the morphology changed significantly postoperatively at the corneal incision, including the cell absent area, wave-like area, dot-like and mass-like hyperreflection, stripe-like absent area, in the endothelial layer, and the keratocyte activation, microfolds, irregular hyporeflective or hyperreflective belt, and a little dot-like hyperreflection in the posterior stroma layer. 3 The reduction of the endothelial cell density at the corneal incision at 1 week, 2 weeks, 1 month postoperatively, were positively correlated with SIA (P1 week = 0.003, P2 weeks = 0.003, P1 month = 0.032), while others were not associated with SIA statistically.

**Conclusions:**

The reduction of endothelail cell density and the histocytological changes at the corneal incision were associated with SIA. The underlining mechanism needs further study.

**Electronic supplementary material:**

The online version of this article (doi:10.1186/1471-2415-14-134) contains supplementary material, which is available to authorized users.

## Background

The aims of modern cataract surgery are the best uncorrected visual acuity, minimal postoperative astigmatism, and rapid visual rehabilitation [1]. Surgical induced astigmatism (SIA) was one of the factors that influences the desirable refractive outcome. SIA was related to the length, type, location, structure of the incision and to the suture closure technique, etc. [1]. The injury of the surgery caused significant changes of endothelial cell and keratocyte especially in cell density [2], function and tissue morphology [3]. The association between the corneal histocytological changes with SIA has not been reported. The association between histocytological changes with SIA has not been reported. Confocal microscope through focusing (CMTF) is a non invasive and relatively easy way to observe the endothelial cell, keratocyte and morphological changes of various corneal layers [4]. The purpose of this study was to investigate the association between histocytological changes of the cornea and SIA.

## Methods

This study enrolled 68 patients (68 eyes) who had underwent phacoemulsification and implantation of a foldable intraocular lens through a clear corneal incision at AHMCQU from October 2011 to April 2012. The mean patient age was 65.12 years ± 9.66 (SD) (range 45 to 83 years), 35 patients were women. Inclusion criteria included visually significant cataract with grade III nucleus sclerosis according to the Emery classification. Exclusion criteria included patients with irregular astigmatism preoperatively, previous ocular surgery or diseases, diabetic or other systematic disease history, and those who cannot undergo Galilei topography measurement due to various causes. Written informed consent was obtained from each patient.

### Clinical examination

The preoperative evaluation included monocular uncorrected visual acuity (UCVA) and best-corrected visual acuity (BCVA), intraocular pressure (IOP) determination, refraction using autorefractometry, ocular axial length measurement and IOL (intraocular lens) spherical power calculation, slitlamp examination, routine fundus examination, and Galilei topography measurement. Astigmatism values and axis were obtained by Galilei topography measurement (SIS Surgical Instrument Systems Ltd. Switzerland). SIA was calculated by Jeffe’s vector analysis.

### Confocal microscope through focusing

The CMTF (Confoscan4, NIDEK Co., Ltd., Japan) measurement and image analysis were performed by the same experienced ophthalmologist before and after 1 week, 2 weeks, 1 month, 3 months and 6 months after phacoemulsification. The CMTF was equipped with a 40 × objective lens. After a topical oxybuprocaine eyedrop was installed in the detected eye, an eye speculum was placed. Then a drop of Carbomer (BauscH&LomB, America) was applied to the objective lens as a coupling agent between the applanating lens cap and the cornea. The lens was advanced manually until the gel contacted the central surface of the cornea, and keep about 1.98 mm between the lens and cornea surface. All confocal scans were depending on full-thickness and full-auto scan mode, the cornea was scanned from the endothelium to the epithelial surface three times and each scan obtaining 350 digital images finally. Each digital image represented a coronal section of 460 × 345um, magnification of 500 ×. Three clear representative images per time were selected for analysis for each eye and averaged. Then the patient gazed upward or downward to keep the incision or contralateral incision corneal plane paralleled with the lens plane, with the same method obtained the images of incision and the contralateral incision. Layers included in the analysis were endothelium and posterior stroma layer (each posterior stroma layer image was apart 40um from endothelium). Endothelial cell density (cells/mm^2^) were calculated using Confoscan 4 NAVIS analysis software automatic count mode, and keratocyte density of posterior stroma layer were counted by its manual count mode.

### Surgical technique

All operations were performed by the same expert surgeon using phacoemulsification with superotemporal incisions in the right eyes and superonasal incisions in the left eyes under topical anesthesia with oxybuprocaine (Santen Co.,Ltd., Japan) dropped three times before surgery. The pupil was fully dilated with tropicamide (Xingqi Co., Ltd., China). The clear corneal tunnel incisions were made 1.0 mm apart from the limbus with a 3.0-mm metal keratome. Phacoemulsification was performed by the Alcon Infiniti system(Alcon Co., Ltd., America), the lens nucleus was removed via phaco chops methods and cortex was removed by automated irrigation and aspiration methods. A foldable intraocular lens was injected into the capsular bag. The viscosurgical device was removed with irrigation and aspiration system. The eyes were filled with balanced salt solution to keep a normal pressure. All incisions were left sutureless, and all wounds were watertight at the end of the surgery. None of the patient experienced significant corneal edema, increased IOP and other complications. Postoperatively, each patient received the same treatment, tobramycin 0.3%-dexamethasone 0.1% (TobraDex®, Alcon) one drop 4 times a day plus ofloxacin 0.3% one drop 4 times a day, and the regimen was tapered over the first postoperative month. Each patient was performed clinical examination include visual acuity, IOP determination, slitlamp examination, Galilei topography and CMTF measurement 1 week, 2 weeks, 1 month, 3 months and 6 months after the surgery.

### Statistical analysis

Statistical analysis was performed using SPSS version 17.0 (SPSS, Inc.), probability values < 0.05 were considered statistically significant. All data were evaluated normality using K-S test, and were presented as means ± standard deviations (SD). ANOVA for repeated measures was used to perform the Statistical data analysis. The least significant difference test was used to calculate statistical differences between two different locations. The Pearson correlation was used to assess the associations between changes in endothelial cell density, keratocyte density of posterior stroma layer and SIA.

## Results

From preoperatively to 1 week, 2 weeks, 1 month, 3 months and 6 months postoperatively, the endothelail cell density at the three locations was decreased significantly (p < 0.05, Additional file 1). At the contralateral incision, it showed less decreased than the other two locations (P_centre-contralateral_ = 0.012, P_contralateral-incision_ = 0.046, Additional file 1). At the incision, it decreased higher with significant difference with that at the contralateral incision(P = 0.046), however, no significant difference was found at the incision and the central cornea (P = 0.596, Additional file 1). The keratocyte density of posterior stroma layer was increased significantly at 1 week, 2 weeks, 1 month, 3 months postoperatively (p < 0.05, Additional file 1), but not statistically significant (p = 0.173, Additional file 1) at 6 months postoperatively. No significant difference was found between any two different locations (p > 0.05).

**Figure 1:**
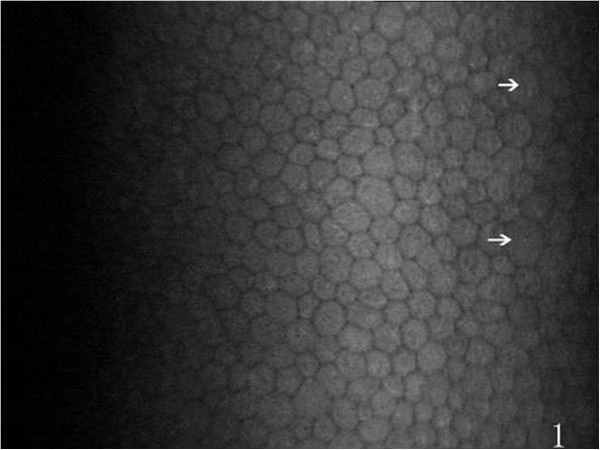
**Preoperative normal corneal endothelium, hexagonal cells uniformly distributed like honeycomb are visible, and a few irregular cells (white arrows) are occasionally seen.**

**Figure 2:**
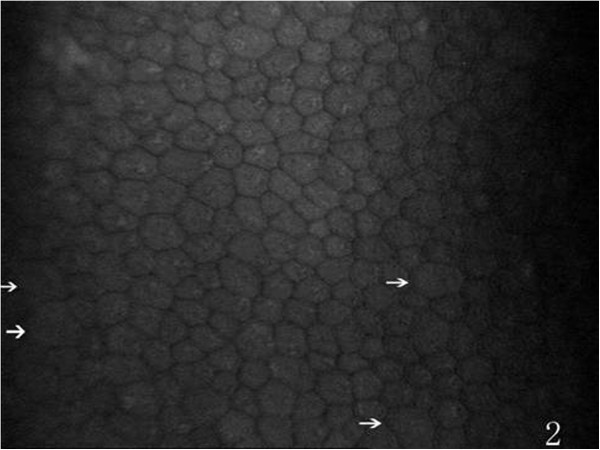
**Postoperatively, the morphology of contralateral incision endothelium, more irregular cells (white arrows) are present (compare with Figure** 1 **).**

**Figure 3:**
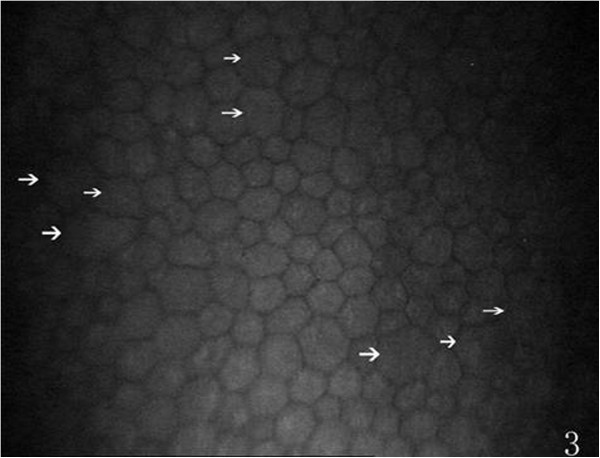
**The morphology of incision endothelium, more irregular cells (white arrows) are present (compare with Figure** 1 **).**

**Figure 4:**
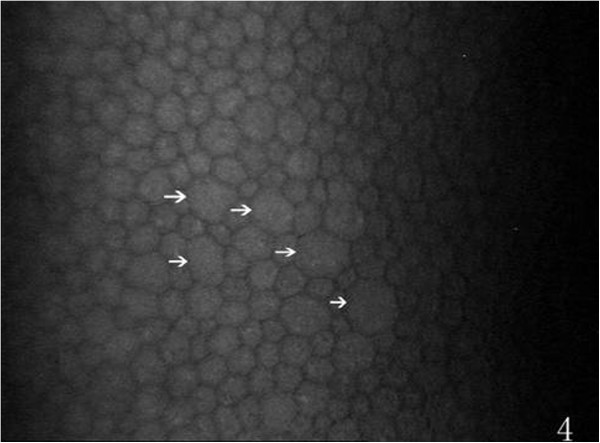
**The morphology of central endothelium, more irregular cell (white arrows) are present (compare with Figure** 1 **).**

**Figure 5:**
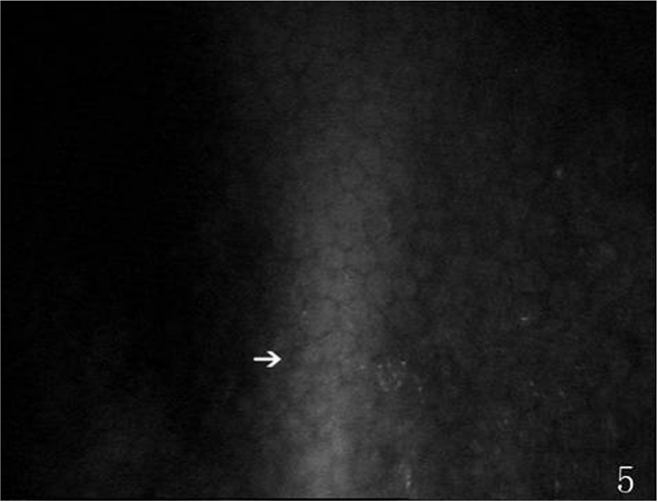
**Showing a wave-like area (white arrow), postoperatively.**

**Figure 6:**
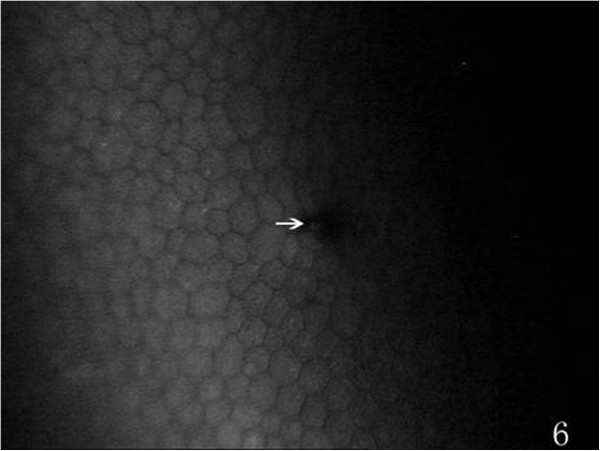
**Showing a endothelial cell absent area (white arrow), postoperatively.**

**Figure 7:**
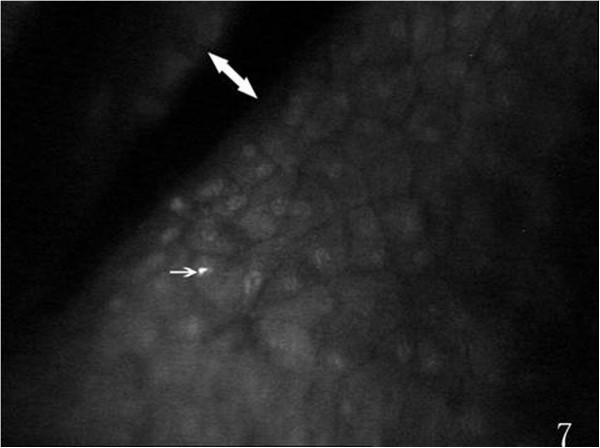
**Showing stripe-like (white double arrow) absent area and dot-like(white arrow), postoperatively.**

**Figure 8:**
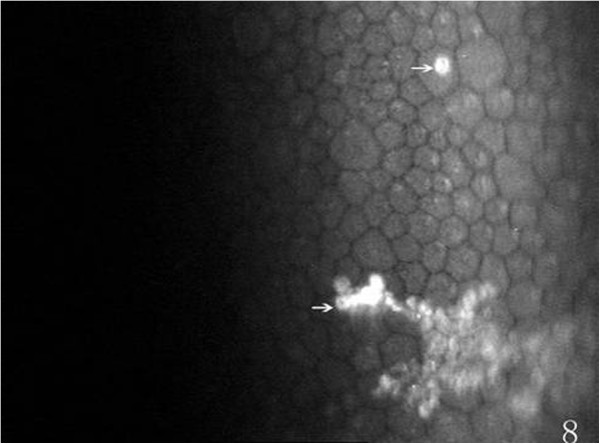
**Showing dot-like and mass-like hyperreflection (white arrows), postoperatively.**

On the endothelial layer preoperatively, the hexagonal cells were uniformly distributed like honeycomb with lightly reflective cytoplasm and clearly defined black cell border (Figure 1), cell nucleus was not visible. Postoperatively, the mean endothelial cell size became much larger, and the size variation among the cells was more obvious. The number of the irregular endothelial cells increased significantly especially after 1 week, 2 weeks and 1 month of the surgery. These changes were more evident at the incision than at the central cornea and contralateral incision (Figures 2, 3 and 4). Postoperatively, wave-like area was visible at the endothelium layer of the incision after 1 week (4 eyes), 2 weeks (2 eyes) and 1 month (1 eye) of the surgery (Figure 5). Endothelial cell absent area was visibal at 1 week (8 eyes), 2 weeks (6 eyes), 1 month (5 eyes), 3 months (3 eyes) and 6 months (1 eye) postoperatively, with more evident than the central cornea and contralateral incision (Figure 6). Besides, stripe-like absent area and dot-like hyperreflective were visible frequently at the incisional endothelium (Figure 7). Postoperatively 1 week (12 eyes), 2 weeks (9 eyes), 1 month (5 eyes), 3 months (3 eyes) and 6 months (2 eyes), dot-like or mass-like hyperreflective were significant obvious at the endothelium (Figure 8).

**Figure 9:**
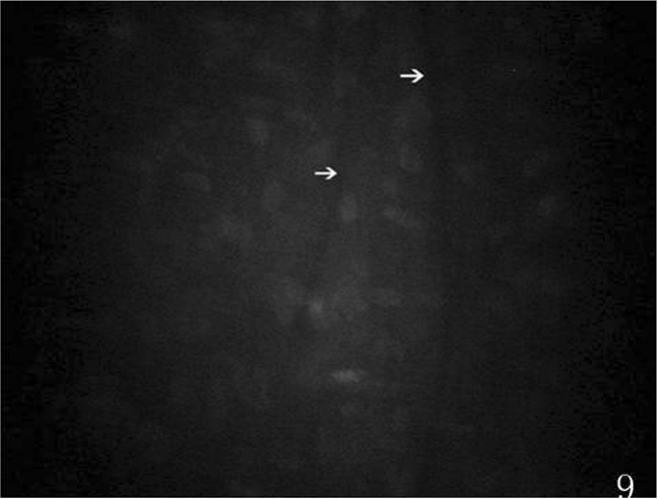
**Showing microfolds (white arrows) of posterior stroma layer, preoperatively.**

**Figure 10:**
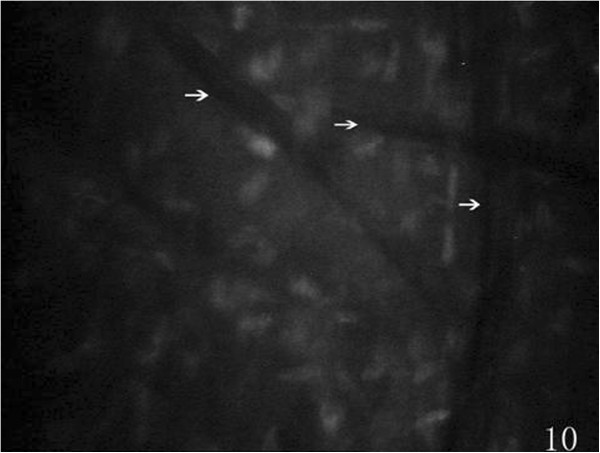
**Showing microfolds (white arrows) of posterior stroma layer, postoperatively.**

**Figure 11:**
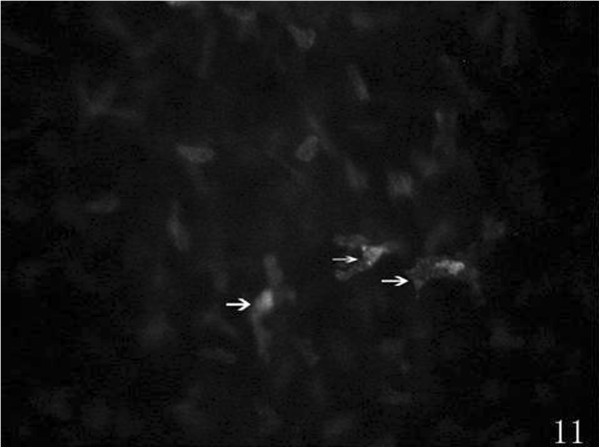
**Postoperatively, activated keratocytes presenting with bright nucleu (white arrows).**

**Figure 12:**
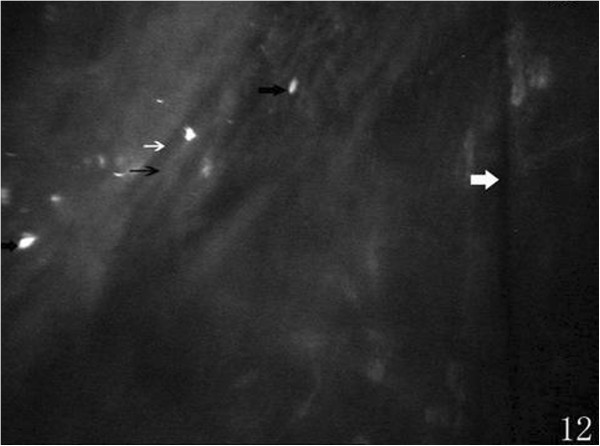
**Postoperatively, posterior stroma layer of incision showing hyporeflective belts (thin white arrow), hyperreflective belts (thin black arrow), dot-like hyperreflection (thick black arrows), and microfold(thick white arrow).**

Preoperatively, at the posterior stroma layer, oval-shaped hypereflective keratocyte nucleus uniformly distributed were visible, cytoplasm and cell boundaries were not visible. Slender hyporeflective dark grain (12 eyes) in the posterior stroma layer was visible, mostly vertical or diagonal arranged preoperatively (Figure 9). At 1 week (38 eyes), 2 weeks (30 eyes), 1 month (23 eyes), 3 months (18 eyes) and 6 months (13 eyes) after the surgery, hyporeflective dark grain around the incision was more obvious than central cornea and contralateral incision (Figure 10). Keratocyte activation was visibal after 1 week (30 eyes), 2 weeks (23 eyes), 1 month (15 eyes), 3 months (6 eyes) and 6 months (4 eyes) postoperatively. Activated keratocyte’s best characterized are bright nucleus, and its size was larger, cytoplasm reflective enhanced and cloudy-like around the nucleus (Figure 11). Postoperatively, disorder arranged hyporeflective or hyperreflective belts were visible at the posterior stroma layer of the incision, dot-like hyperreflective and hyporeflective dark grain were visible around the incision (Figure 12).

The reduction of endothelial cell density on the incision was positively correlated with SIA at 1 week, 2 weeks, 1 month (P_1week_ = 0.003, P_2weeks_ = 0.003, P_1month_ = 0.032) postoperatively, the others were not associated with SIA statistically. However, no significant correlation was found between the increase of the keratocyte density of posterior stroma layer with SIA.

## Discussion

Phacoemulsification has become the main treatment method of cataract, less tissue damage and rapid postoperative rehabilitation are the aims of modern cataract surgery. Several factors are related with the cornea damage induced by the surgery, including phacoemulsification time and energy, the degree of nucleus sclerosis, the depth of the anterior chamber, pupil size, patient age, infusion fluid used and preoperative endothelium function, etc. [5]. After injury, the first observable change of stroma was keratocytes around the cut edge begin to fragment and undergo cell death [6]. About 6 hours post-injury, the adjacent keratocytes then begin to lose their quiescence and become activated [6]. The response to the surgical injury of the keratocyte in the posterior stromal layer is more representative, because it is much closer to the anterior chamber. We choose it as the object in this study.

In this study, the postoperative endothelial cell density decreased significantly, and the loss percentage of endothelial cell was about 12.41% at 6 months after surgery, and it was similar to the report of Walkow T [7]. The reduction of the endothelial cell density disordered the corneal endothelial barrier and pump function. As a result, corneal edema was produced. The endothelial cell density decreased significantly lower at the contralateral incision than at the other two locations, which showed the endothelium at this location suffered less damage. The endothelial cell density of the incision decreased higher, but there was no significant difference between the incision and the central cornea. The possible reason was that the central endothelium suffered mechanical trauma of lens fragments and phaco power damage, the endothelium of the incision suffered mechanical trauma of surgical instruments and infusion fluid impact, while at the contralateral incision, there were no such mechanical trauma and less phaco power damage. From the CMTF images we observed the hexagonal endothelial cells decreased and the variation of the cell size was obvious. These changes in the endothelial cell morphology showed that the normal function was impaired, and the barrier and pump function of the endothelium also damaged. Especially, the changes of the endothelial cell morphology at incision were more obvious than that of the other two locations. Some of the CMTF images showed endothelial cell absent area and stripe-like absent area of incision, and this should be one of the causes of postoperative corneal edema around the incision. 6 months postoperatively, endothelial cell absent area was still visible in one patient, demonstrated that the endothelial cell healing procedure was slow. Meanwhile, the SIA of this patient was greater than the others, this prompted that serious endothelial cell death induced greater SIA. The serious endothelial cell death caused more evident corneal edema, then resulted in greater changes of corneal curvature, thereby forming larger SIA. At the margin of the incision, wave-like area was visible and increasingly rare at endothelium, this probablely induced by corneal edema. Dot-like or mass-like hyperreflection was found on endothelium which need to be further studied. Endothelial cell density reduction, changes of morphology and formation of endothelial cell absent area postoperatively may induce corneal edema, especially at the incision. Corneal edema caused the change of corneal curvature, thereby affected the SIA. With the cell migration and the barrier function of endothelium restored gradually, the edema relieved, so did the SIA.

The keratocyte density of the posterior stromal layer was increased significantly at 1 week, 2 weeks, 1 month, 3 months postoperatively. The highest level was at 1 month, and returned to preoperative level at 6 months. The earliest stromal event after epithelial injury was keratocyte death [8], and an increasing proportion of keratocyte die by necrosis as the wound healing response continues [9]. About 6 hours post-injury keratocyte lose their quiescence and transformed to the activated phenotype [6]. About 3–6 days was the most activated phenotype, and after 2 months activated keratocyte become to the quiescence gradually [10]. These research [6, 8, 9] explains the results of our study. Keratocyte density of 1 week postoperatively was lower than that of 1 month, this was probably related to keratocyte death after injury and corneal edema was serious at postoperative 1 week. Additionally, the patients mean age was 65.12 years ± 9.66, lower keratocyte activity may also affected the measuring results. Postoperatively, no significant difference was found between any two different locations, showed changes of postoperative keratocyte density didn’t appear difference on the location. The changes of morphology in posterior stroma layer was visibal. Preoperatively, 12 eyes existed slender hyporeflective dark grain in the posterior stroma layer, it also named microfolds. Although investigations about those microfolds were limited, some studies reported that it was positively correlated with stromal edema [11, 12]. This was similar to Hollingswo’s [13] report that the microfolds incidence of 70 years old volunteers was 18%. We observed 13 eyes still existed microfolds postoperative 6 months, significantly reduced than 1 week, and reduced to preoperative incidence. Therefore, we speculated that the formation of microfolds due to corneal edema caused by postoperative endothelium dysfunction. Meanwhile, microfolds around the incision was obvious than central cornea and contralateral incision, this was coincide with postoperative serious corneal edema of incision. Postoperatively, keratocyte activation was visibal, many studies reported that the activation of keratocyte was relate to corneal trauma, inflammatory response etc. [14, 15]. After phacoemulsification, keratocyte activation was induced by surgical stimulation and anterior segment inflammatory reaction. Those activated keratocyte could efficiently synthesize extracellular matrix components such as collagen and proteoglycans [16]. Gradually, deposition of fibrotic repair tissue transformed to scar, those repair tissue was opaque and its contraction alters corneal shape [6], interfering with the corneal curvature. We concluded the disorder arranged hyporeflective or hyperreflective belts were incision scars, and postoperative residual astigmatism was causes by those scars.

The reduction of endothelial cell density at the incision was positively correlated with SIA at 1 week, 2 weeks and 1 month postoperatively, and the changes of the another two locations were not associated with SIA statistically. The more serious the endothelial cell was injuried, the more obvious of corneal edema at the incision, which caused the more changes of corneal curvature, and resulted in more SIA. However, there was no significant correlation between keratocyte density of posterior stroma layer and SIA. This showed the keratocyte density increase didn’t result in the changes of corneal curvature.

## Conclusions

In summary, after phacoemulsification, keratocyte, endothelial cell and corneal tissue morphology changed significantly. In addition to the incision length, position, shape, structure, sutures and other factors, we observed that the reduction of endothelial cell density at the incision also affect the SIA. Therefore, more attention should payed to protect the endothelial cells during cataract surgery.

## Electronic supplementary material

Additional file 1: Table S1: The changes of endothelial cell density and keratocyte density of posterior stroma layer ( ±s,n=68,cells/mm^2^). **Table S2.** The mean SIA at different time ( ±s)D,n=68. **Table S3.** The association analysis of the changes of endothelial cell or keratocyte density and SIA. **Figure S1.** The scatter plot of endothelial cell density reduction on incision and SIA at 1 week, 2 weeks, 1 month postoperatively. (DOC 186 KB)
