# Supplementary material for: Preliminary study of the association between corneal histocytological changes and surgically induced astigmatism after phacoemulsification
Source: BMC Ophthalmol. 2014 Nov 20;14:134. doi: 10.1186/1471-2415-14-134 (PMC4256752; doi:10.1186/1471-2415-14-134)
Supplement: Supplementary file 1 — Additional file 1: Table S1: The changes of endothelial cell density and keratocyte density of posterior stroma layer ( ±s,n=68,cells/mm2). Table S2. The mean SIA at different time ( ±s)D,n=68. Table S3. The association analysis of the changes of endothelial cell or keratocyte density and SIA. Figure S1. The scatter plot of endothelial cell density reduction on incision and SIA at 1 week, 2 weeks, 1 month postoperatively. (DOC 186 KB) [file 12886_2013_507_MOESM1_ESM.doc]

Additional file 1

Table 1 The changes of endothelial cell density and keratocyte density of posterior stroma layer (
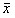
±s，n=68，cells/mm^2^)

| endothelial cell keratocyte |
| --- |
| Time incision central cornea contralateral incision incision central cornea contralateral incision |
| preop 2642.00±358.603 2586.71±345.809 2670.65±354.363 738.63±108.767 748.13±101.490 741.10±102.129  postop1week 2445.12±350.415 2410.93±351.169 2542.97±357.819 763.66±107.583 771.28±100.571 769.22±103.262  postop2weeks 2375.66±351.808 2347.10±350.930 2499.96±356.910 773.69±109.103 781.65±101.796 778.24±103.567  postop1month 2322.35±362.520 2295.99±355.678 2467.78±360.008 806.81±107.910 813.49±101.010 807.51±102.162  postop3months 2280.22±351.420 2254.60±355.028 2446.43±362.546 764.75±107.546 769.26±102.669 762.24±103.157  postop6months 2258.72±352.547 2235.69±354.234 2427.51±364.524 739.19±109.017 747.66±101.722 741.72±101.977 |
| preop=preoperatively;postop1week=postoperative 1 week;postop2weeks=postoperative 2 weeks;postop1month=postoperative 1 month; postop3months=postoperative 3 months; postop6months=postoperative 6 months. |

Table 2 the mean SIA at different time(
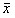
±s)D，n=68

| Time postop 1week postop 2 weeks postop 1month postop 3months postop 6months |
| --- |
| SIA 1.267±0.345 1.034±0.289 0.816±0.248 0.641±0.208 0.626±0.233 |
| postop1week=postoperative 1 week;postop2weeks=postoperative 2 weeks;postop1month=postoperative 1 month; postop3months=postoperative 3 months; postop6months=postoperative 6 months. |

Table 3 The association analysis of the changes of endothelial cell or keratocyte density and SIA.

| location  time | endothelial cell keratocyte |
| --- | --- |
|  | incision central cornea contralateral incision incision central cornea contralateral incision |
| 1week r=0.359 p=0.003 r=0.085 p=0.489 r=-0.008 p=0.945 r=-0.066 p=0.595 r=0.094 p=0.435 r=0.007 p=0.958  2weeks r=0.352 p=0.003 r=0.136 p=0.267 r=0.128 p=0.299 r=-0.014 p=0.911 r=0.013 p=0.918 r=-0.014 p=0.913  1month r=0.260 p=0.032 r=0.050 p=0.683 r=0.105 p=0.396 r=-0.157 p=0.200 r=-0.149 p=0.226 r=-0.033 p=0.789  3months r=0.153 p=0.213 r=-0.038 p=0.757 r=0.040 p=0.748 r=-0.006 p=0.961 r=0.022 p=0.859 r=0.018 p=0.884  6months r=0.148 p=0.228 r=-0.104 p=0.401 r=-0.068 p=0.580 r=0.042 p=0.734 r=0.192 p=0.117 r=0.001 p=0.991 | |
| 1week=postoperative 1 week; 2weeks=postoperative 2 weeks; 1month=postoperative 1 month; 3months=postoperative 3 months; 6months=postoperative 6 months. | |

Figure S1: The scatter plot of endothelial cell density reduction on incision and SIA at 1 week, 2 weeks, 1 month postoperatively.
